# A Multifrequency Millimeter-Wave CMOS Sensor for Non-Invasive Continuous Glucose Monitoring Using UMC 0.18 μm Technology

**DOI:** 10.3390/bios16090460

**Published:** 2026-08-25

**Authors:** Dalia Elsheakh, Ratshih Sayed, Hebatullah H. Draz, Ghada H. Ibrahim, Heba Shawkey

**Affiliations:** 1 Electrical and Basic Science Department, Faculty of Engineering Technology, El Sewedy University of Technology, 10th of Ramadan City 44629, Egypt; 2 Microstrip Department, Electronics Research Institute, El Nozha 11843, Egypt; 3 Microelectronics Department, Electronics Research Institute, El Nozha 11843, Egypt; ratshih@eri.sci.eg (R.S.); hdraz@eri.sci.eg (H.H.D.); ghamdy@eri.sci.eg (G.H.I.); heba_shawkey@eri.sci.eg (H.S.)

**Keywords:** millimeter-wave (mm-wave), wireless communication, W band, high-frequency wireless systems, integrated antenna, multi-arm (MA), UMC 180 nm CMOS

## Abstract

Diabetes is a major worldwide health concern, which emphasizes the critical need for precise and continuous glucose monitoring devices. This paper introduces a novel, non-invasive method for continuous blood glucose monitoring using on-chip multi-arm sensors designed as earbuds by using UMC 0.18 μm technology. The proposed sensor uses the dielectric characteristics of the earbud to detect variations in glucose levels while operating at various resonant frequencies, including 32, 42, 64, and 94 GHz. The sensitivity of the proposed method was evaluated using a reflection coefficient criterion of $|S_{11}|\leq -6\;\mathrm{dB}$, confirming its ability to achieve accurate detection when implemented within an earbud device. A 3D electromagnetic high-frequency structure simulator (HFSS) is used to validate the simulation. Only |S_11_| data are used to determine the glucose concentrations in the blinded prediction group. The results demonstrate a strong correlation between sensor responses and glucose levels. The sensor achieved a sensitivity of 12.4 MHz/mg/dL, 6 dB/mg/dL. Moreover, the earbud’s homogeneous tissue architecture and naturally low eccrine sweat gland density lessen susceptibility to confounding physiological variables commonly observed in microwave-based glucose detection. As a major advancement in biomedical sensing technology, this wearable system provides a precise and useful method for non-invasive glucose monitoring.

## 1. Introduction

Technological developments have improved medical diagnoses, treatments, and procedures over time, as well as patient quality of life [1,2,3]. Diabetes mellitus (DM) sensing and monitoring has been the subject of various efforts in this respect [4]. It is predicted that there will be 366 million instances of diabetes mellitus (DM) in 2030 [5,6]. High blood glucose levels (BGLs), often known as persistent or chronic hyperglycemia, are a hallmark of a group of metabolic disorders collectively referred to as diabetes [7]. Chronic exposure to hyperglycemia is known to cause damage to the kidneys, nerves, eyes, and other organs, as well as cardiovascular problems [8,9,10,11,12].

**Figure 1:**
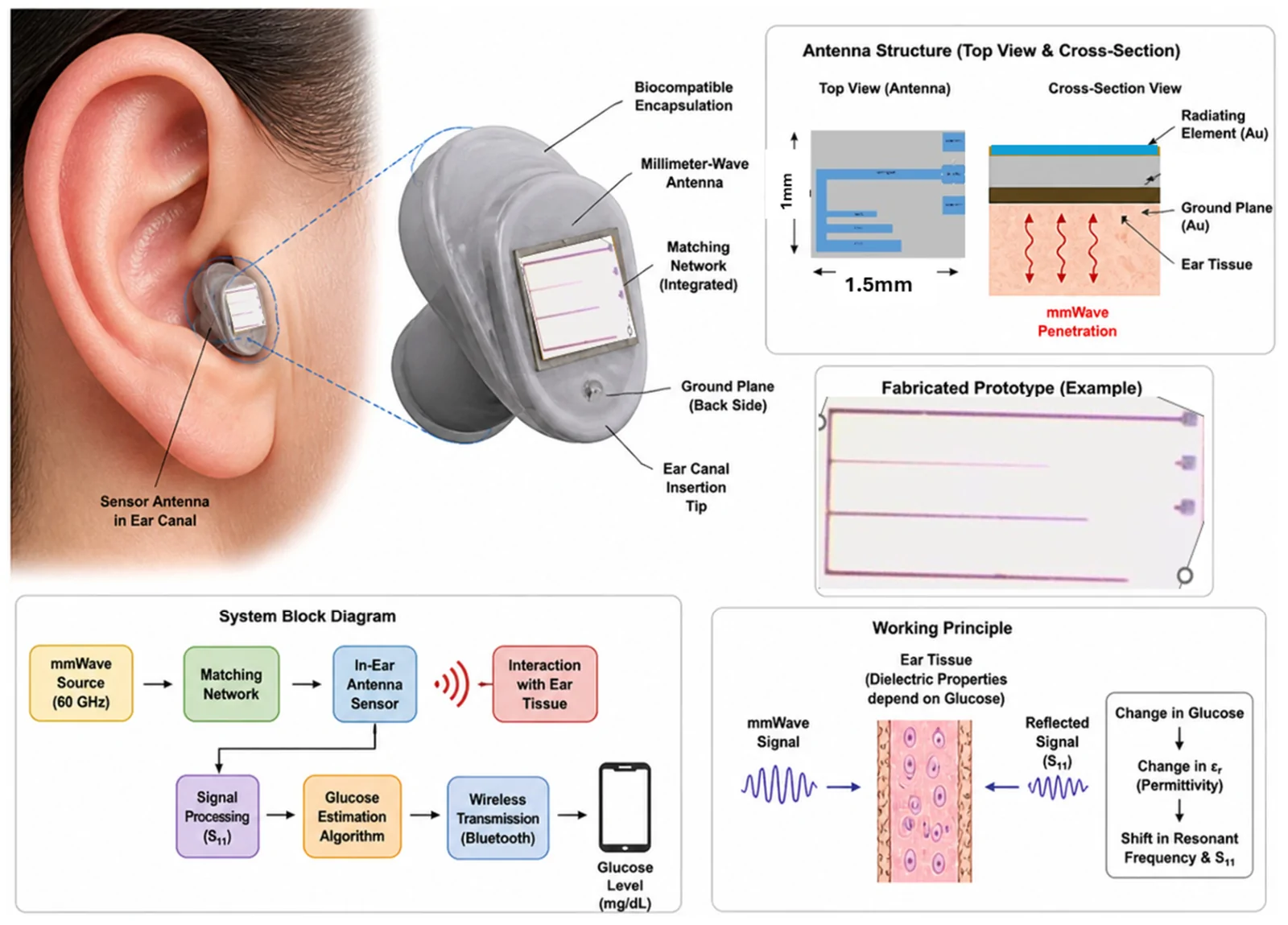
The proposed system.

Microwave glucose sensing frequency choices fall into three useful bands for taxonomy and design: 1–3 GHz, 3–6 GHz, and >6 GHz. Band selection determines sensitivity trade-offs, EM field confinement, and penetration depth; lower bands average larger tissue volumes and penetrate deeper; mid bands >6 GHz provide intermediate behavior (including mm-wave) and concentrate fields close to the skin’s surface, making it ideal for fingertip or superficial vessel sensing [13]. The rapid growth of data-intensive applications and next-generation wireless systems has driven increasing interest in the millimeter-wave (mm-wave) spectrum, typically defined within the frequency range of 30–300 GHz. Among these, 32 GHz, 42 GHz, 64 GHz, and 94 GHz have emerged as key candidates for enabling high-capacity communication, advanced sensing, and emerging Sixth-Generation (6G) technologies, as shown in Figure 1. These frequency bands offer significantly larger bandwidths compared to conventional microwave frequencies, thereby supporting ultra-high data rates, low latency, and high-resolution imaging capabilities [14,15]. The 40 GHz band, located within the lower mm-wave range and partially overlapping with the Ka band, has been widely adopted in fixed wireless access, satellite communications, and cellular backhaul networks. Its relatively moderate atmospheric attenuation and favorable propagation characteristics make it suitable for medium-range, high-capacity links, particularly in dense urban environments [16].

The 60 GHz band has attracted considerable interest because of its unlicensed spectrum and wide contiguous bandwidth, making it suitable for short-range, high-speed wireless communication systems such as Wi-Fi (IEEE 802.bd). It supports multi-gigabit indoor applications including virtual reality, high-definition multimedia streaming, and wireless personal area networks. Although oxygen absorption at 60 GHz limits propagation distance, it reduces interference and improves spatial reuse in dense environments [17]. Millimeter-wave (mm-wave) and microwave (MW) frequencies are widely used in dielectric spectroscopy for non-invasive glucose sensing because they can penetrate deep tissue layers more effectively than optical methods. Glucose molecules interact with electromagnetic waves through reflection, transmission, resonant vibration, and radar backscattering depending on the operating frequency, enabling accurate glucose detection. The main interaction mechanisms include reflection, transmittance, resonant perturbation, and radar sensing, with many reported studies operating in the 1–10 GHz microwave range [18,19,20,21].

To reduce the effect of sample position variations, which can cause amplitude shifts up to 1.08 dB, Lee et al. [22] employed wideband 0.5–18 GHz spectra (S_11_/S_21_) with 401 points combined with a 1D CNN (convolution neural network) model. Their work demonstrated that full-spectrum multi-band features improve robustness against placement variability without requiring additional calibration or rigorous cross-validation. Millimeter-wave (mm-wave) and microwave (MW) sensing techniques offer high sensitivity, fast real-time response, flexibility, low power consumption, compact size, portability, robustness, cost-effectiveness, and easy fabrication without requiring precise alignment [23,24,25]. The mm-wave spectrum ranges from 30 to 300 GHz, with the W band (75–110 GHz) showing strong potential for in vivo glucose monitoring through skin tissue [26,27,28]. However, continuous exposure to mm-wave and MW signals may cause tissue damage because of their penetration depth [29].

Recent studies have demonstrated promising non-invasive glucose monitoring approaches using handheld mm-wave devices through the earlobe, finger web, fingertip, compact finger-slot readers, and forearm [30,31,32,33]. These studies showed that skin, fat, and blood significantly influence the reflected signal, whereas muscle and bone have minimal effects [34]. In addition, Shaker and Smith [35] investigated a 60 GHz retrofitted Soli alpha radar system and demonstrated that variations in dielectric properties could be detected by optimizing the transmit and receive antenna configuration. Their results highlighted the feasibility of integrating this technology into wearable finger pulse oximeter devices, although physiological blood variations still require further calibration and mapping studies.

Like dielectric spectroscopy, radio frequency (RF) spectroscopy analyzes the interaction between electromagnetic waves and biological tissues to estimate glucose concentration [35,36]. RF spectroscopy commonly operates within the 0.1–20 GHz range and detects resonant frequency shifts using resonator sensors such as interdigital transducers (IDTs) and stepped impedance resonators (SIRs) [37]. Yunos and Manczak [37] demonstrated a linear relationship between glucose concentration and resonance frequency shifts using RF sensors, highlighting their suitability for continuous non-invasive glucose monitoring. Changes in glucose concentration alter tissue dielectric properties, directly affecting the RF signal while providing a rapid and cost-effective sensing approach [35].

Using multifrequency operation at high frequencies provides a significant advantage for non-invasive glucose monitoring because biological tissues exhibit complex, frequency-dependent dielectric behavior. At microwave and millimeter-wave ranges (30–110 GHz), electromagnetic waves interact with tissue through mechanisms such as dipolar relaxation, ionic conduction, and bound water polarization, all of which are subtly influenced by glucose concentration. A single frequency often cannot isolate glucose effects from other physiological variables like temperature, hydration, or tissue heterogeneity. In contrast, a multifrequency approach captures a spectral “fingerprint” of the tissue response, enabling more robust differentiation between glucose-induced variations and background noise. High frequencies further enhance sensitivity due to their shorter wavelengths and stronger interaction with superficial tissue layers where interstitial glucose dynamics are prominent. By combining measurements across multiple frequencies, advanced signal processing or machine learning models can improve accuracy, stability, and calibration, making multifrequency high-frequency sensing a more reliable strategy for continuous, non-invasive glucose monitoring.

Figure 1 presents an infographic illustrating the overall architecture and development roadmap of the proposed non-invasive glucose monitoring system. The complete framework includes sensor design, integration into an earbud platform, signal processing, glucose estimation, and future clinical validation.

This paper focuses on the sensor development stage of the proposed framework. The remainder of the paper is organized as follows. Section 2 describes the design, simulation, fabrication, and performance evaluation of the proposed multi-arm sensor. Section 3 investigates the effects of varying glucose concentrations in the ear phantom on the sensor performance for wearable and implantable applications and presents the corresponding measurement results. Section 4 discusses experimental findings, including the observed frequency shifts under different phantom positions and glucose levels. Finally, Section 5 summarizes the key findings and concludes the paper.

## 2. Materials and Methods

The proposed sensor design was analyzed using the Ansys High-Frequency Structure Simulator (HFSS), version [XX], developed by Ansys, Inc., Canonsburg, PA, USA. HFSS is a full-wave electromagnetic (EM) simulation software widely used for the analysis and design of high-frequency electromagnetic structures and devices.

**Figure 2:**
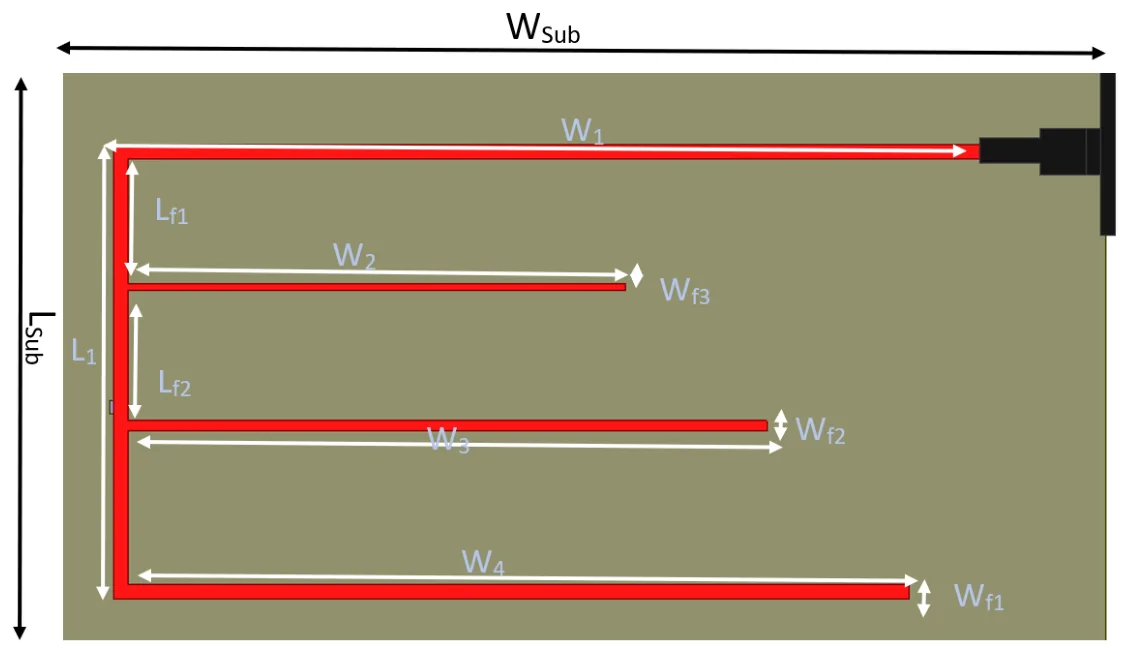
The proposed multi-arm (MA) structure.

This simulator is used to design the proposed sensor. The multi-arm antenna was designed to target UMC 0.18 μm technology and fabricated using the same technology as shown in Figure 2. This technology offers a stack of six aluminum metal layers, where the top metal layer is offered in a thicker option for lower parasitic resistance, providing lower losses in RF and microwave applications [38]. The multi-arm antenna is implemented on the thick top metal (M6, layer thickness of 2 μm), while the ground plane is implemented on the lower metal layer (M1, layer thickness of 1.2 μm). The antenna is first constructed from a rectangular strip as a stub to further add log arms with different lengths and transmission line widths *Lp* as the main resonator according to Equations (1) and (2) [39]; each resonant has its own length stub.

**Figure 3:**
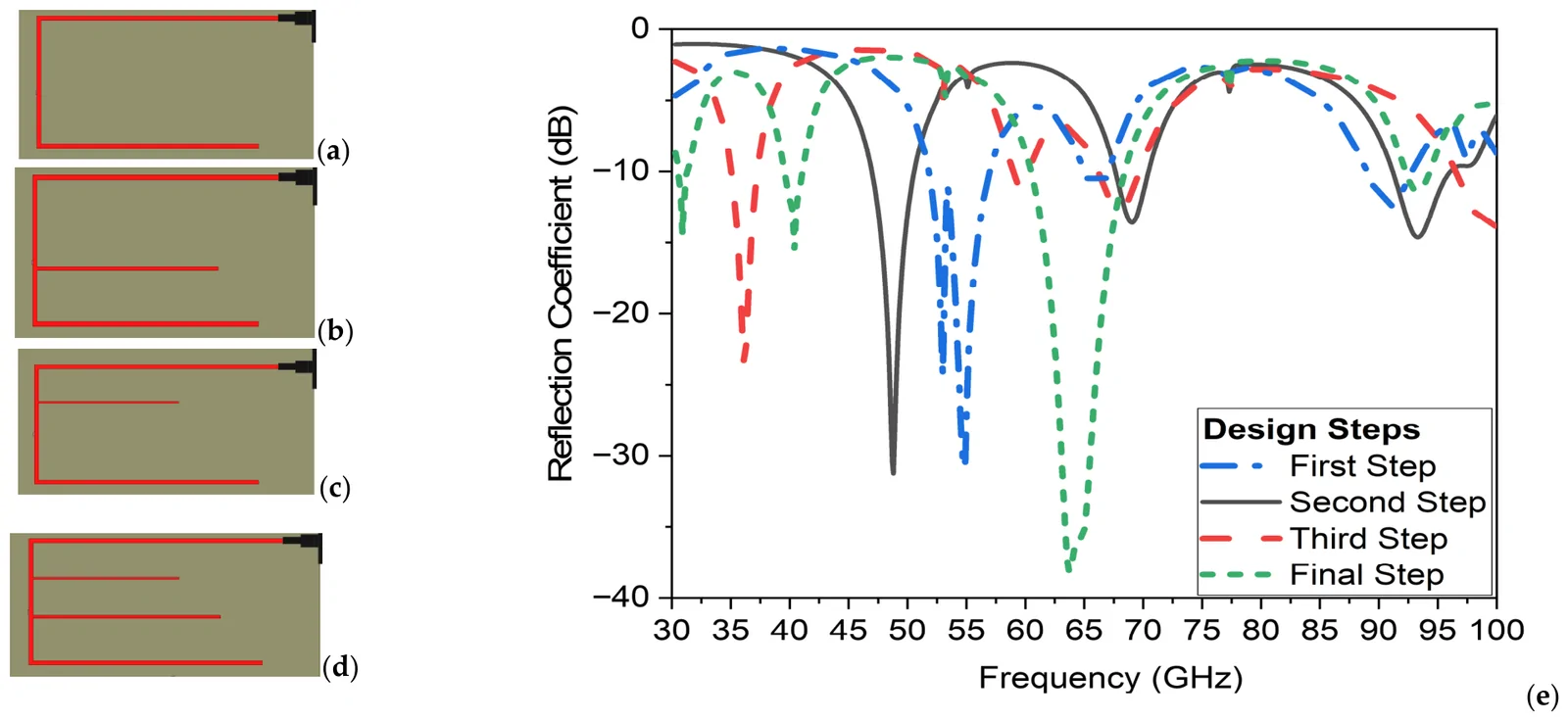
(**a**) First design, (**b**) second design, (**c**) third design, (**d**) final design and schematic of proposed sensor, and (**e**) |S11| for design step.

The length of the main rectangular patch resonator ($L_{p}$) is calculated to achieve resonance at the desired fundamental frequency ($f_{r}$) as shown in Figure 3. This calculation accounts for the effective dielectric constant ($\varepsilon_{reff}$) and the fringing field extension (ΔL) at the patch edges, as shown in Equation (1), where c is the speed of light in free space. The effective dielectric constant depends on the substrate’s permittivity and the patch width.

(1) $$L_{p}=\frac{c}{2f_{r}\sqrt{\varepsilon_{reff}}}-2\Delta L.$$

To implement “log arms” with varying lengths and widths, a log-periodic scaling approach is used as shown in Figure 3a–d. A scaling factor τ is applied to ensure consistent wideband performance; each successive element (arm) is scaled from the previous one by the same factor, as given in Equation (2). This maintains the antenna’s electromagnetic behavior over a broad frequency range, as shown in Figure 3e.

(2) $$\frac{L_{n+1}}{L_{n}}=\frac{W_{n+1}}{W_{n}}=\tau .$$

The four resonant frequencies are determined by distinct path lengths: 32 GHz (fundamental) corresponds to $W_{1}+L_{1}+W_{4}$; 42 GHz (second) to $W_{1}+L_{f1}+L_{f2}+W_{f2}$; 64 GHz (third) to $W_{1}+L_{f1}$; and 94 GHz (fourth) to $W_{1}$. The optimized dimensions of the proposed antenna sensor are tabulated in Table 1.

**Figure 4:**
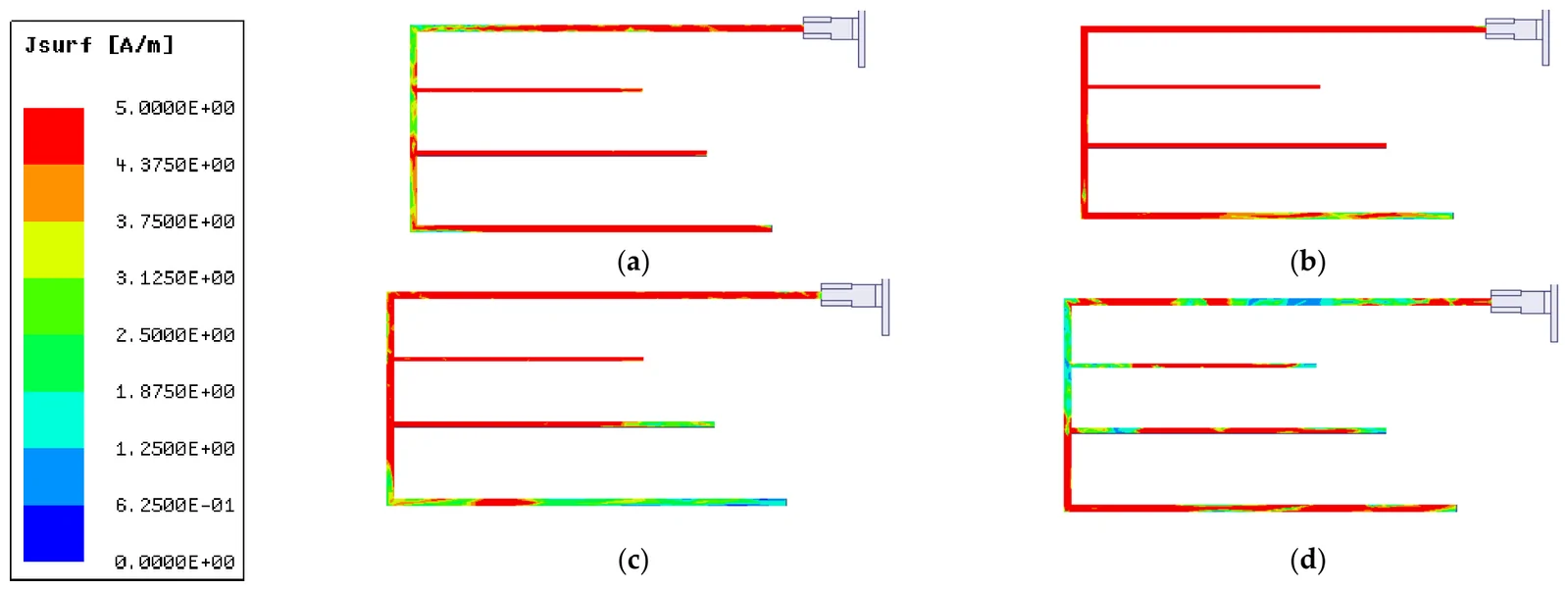
The current distribution of the proposed sensor at four resonant frequencies: (**a**) 32 GHz, (**b**) 42 GHz, (**c**) 64 GHz, and (**d**) 94 GHz.

Figure 4 depicts the current distribution at various resonant frequencies on the surfaces of the two meander lines of the proposed sensor antenna. According to the current distribution pattern, most currents are found along the transmission line and along the largest arm for 32 GHz and the smallest resonator arm for 94 GHz, respectively. The multi-arm resonator arms are the areas where the currents are concentrated.

**Figure 5:**
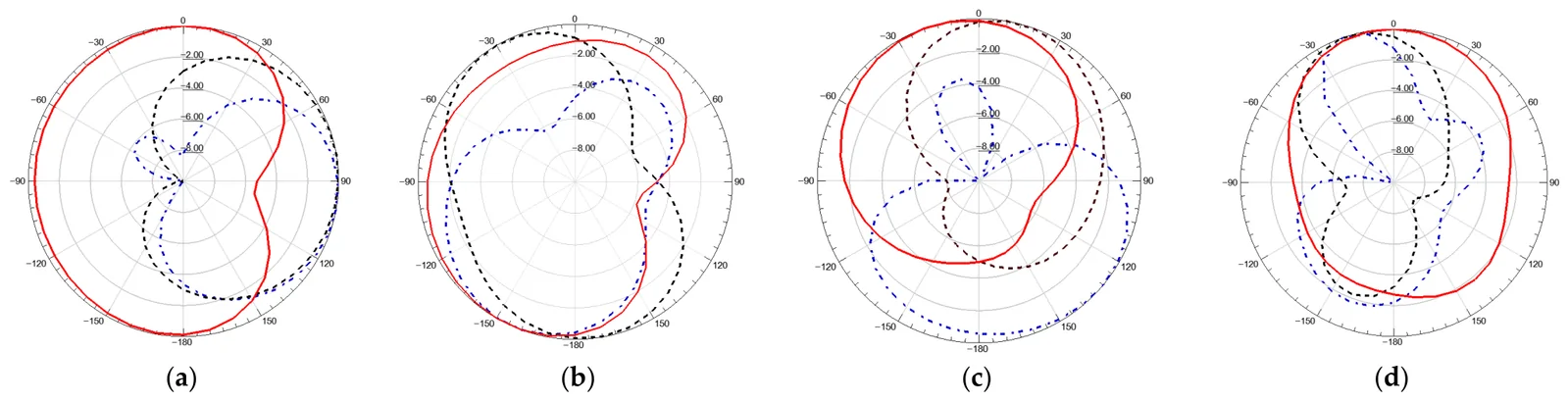
Simulated radiation pattern for the proposed antenna sensor at (**a**) 32 GHz, (**b**) 42 GHz, (**c**) 64 GHz, and (**d**) 94 GHz.

Figure 5 presents the simulated 2D radiation patterns of the proposed antenna sensor at both E and H planes for the xz-, yz-planes and xy-plane at four different frequencies (32 GHz, 42 GHz, 64 GHz, and 94 GHz), respectively. The patterns are shown for three orthogonal planes: the xz plane (red solid line), the yz plane (black dashed line), and the xy plane (blue dash dotted line), representing the E plane and H plane characteristics accordingly. At 32 GHz, the radiation patterns exhibit a relatively broad main beam in all planes, with minor sidelobes and nearly omnidirectional behavior in the xy plane. As the frequency increases to 42 GHz, the main lobes become slightly narrower, indicating an increase in directivity. At 64 GHz, further beam squinting and a reduction in half-power beamwidth are observed, particularly in the xz and yz planes, while the xy plane pattern remains more uniform. Finally, at 94 GHz, the patterns show the highest directivity, with well-defined main lobes and increased sidelobe levels, especially in the xz plane. Overall, the antenna sensor maintains stable radiation performance across the operating band, with frequency-dependent beam shaping that is suitable for multi-band sensing applications.

## 3. Results

This section presents the simulated performance of the proposed multi-arm on-chip glucose sensor, as well as measurement of the proposed sensor.

### 3.1. Proposed Technique for Glucose Levels

**Figure 6:**
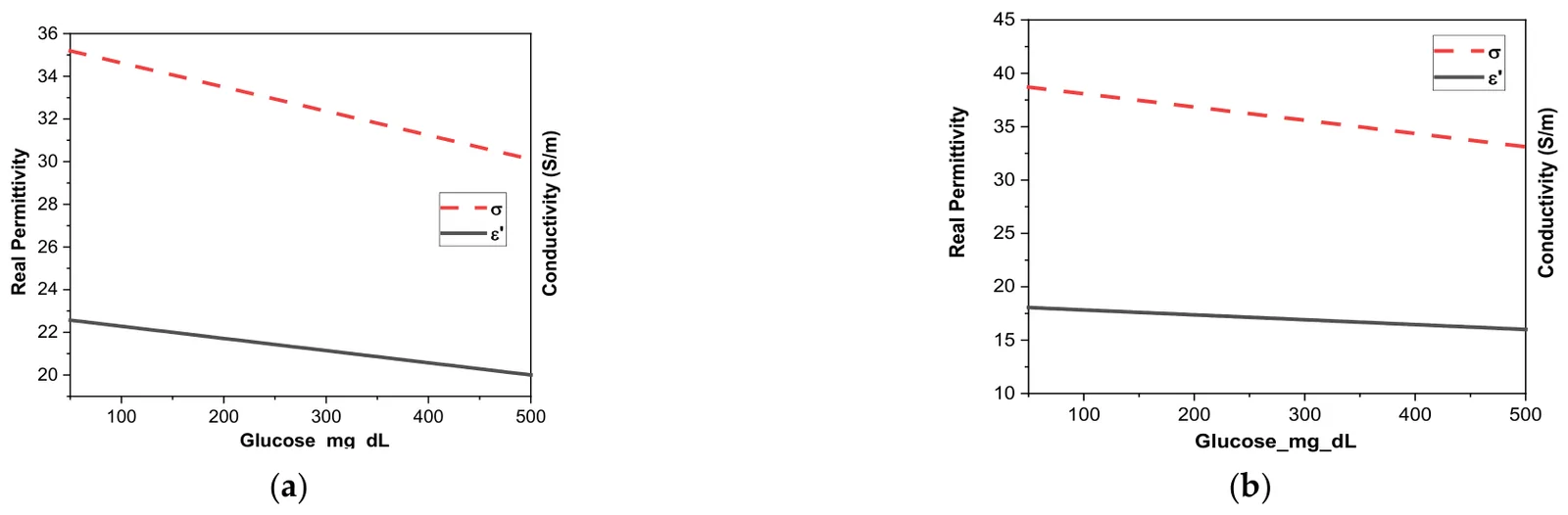

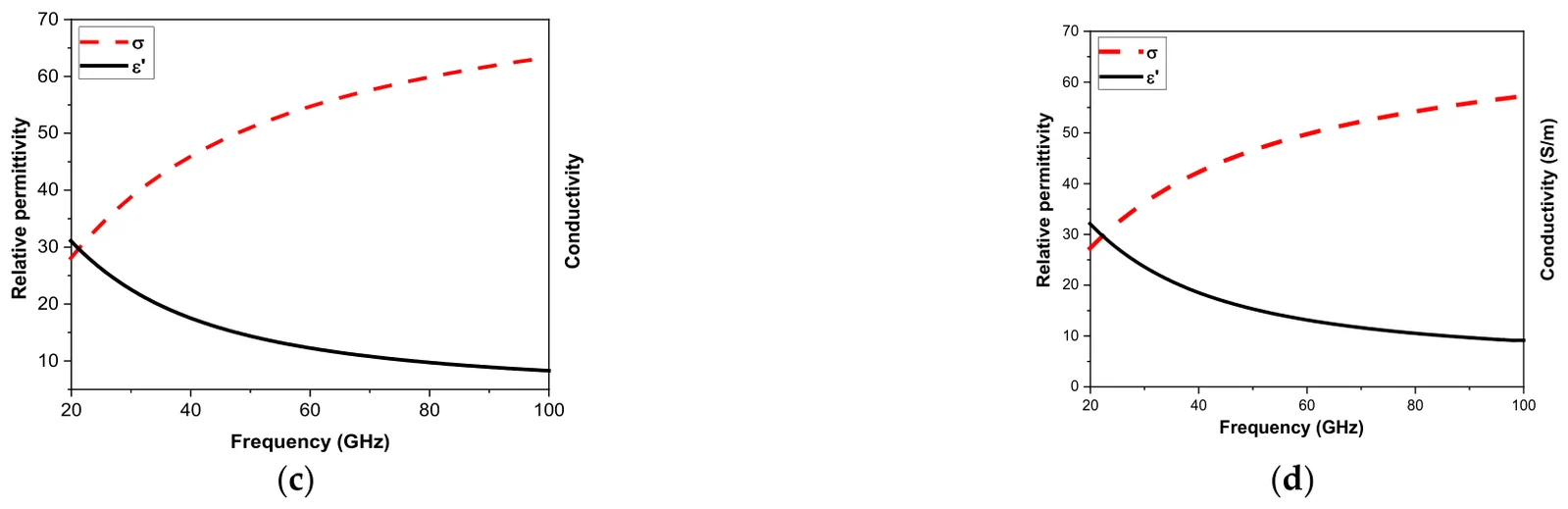
The electrical properties of glucose levels at (**a**) 32 GHz, (**b**) 42 GHz, (**c**) 64 GHz, and (**d**) 94 GHz.

To evaluate the sensitivity of the proposed sensor, it is assumed to be a set of sensors working at different frequency bands. The multi-arm of each sensor is sequentially loaded with a phantom layer, as illustrated in Figure 6. As the electrical properties of the blood and tissue layers are frequency-independent, the Cole–Cole model is used to characterize the complex permittivity of these media, as in [39].

(3) $${\varepsilon_{r}}^{*}(\omega )=\varepsilon_{\infty }+\frac{\Delta \varepsilon }{{1+(j\omega \tau )}^{1-\alpha }}+\frac{\sigma_{s}}{j\omega \varepsilon_{0}},$$

where ε_∞_ is the relative permittivity of the high frequency, Δεn is the pole amplitude, τ denotes the relaxation time, and n represents the number of poles. The Cole–Cole model for complex conductivity is presented in [40].

(4) $$\varepsilon_{r}^{*}={\varepsilon_{r}}^{\prime }+j{\varepsilon_{r}}^{\prime \prime }.$$

(5) $${\varepsilon_{r}}^{\prime \prime }=-\frac{\sigma_{s}}{\omega \varepsilon_{0}}.$$

Equations (4) and (5) display the expression for the complex permittivity ${\varepsilon_{r}}^{\prime \prime }$, which typically characterizes the dielectric characteristics of the tissue. j represents the imaginary unit, σ_s_ represents the static conductivity, and ε_r_′ is the real portion of the permittivity of the tissue [39,40].

The Cole–Cole model can be used to describe the dielectric characteristics of biomaterials throughout a broad frequency range. The 30–100 GHz spectrum is used for the measurements in this study. Consequently, it was anticipated that there would be fewer than four poles while looking for a Cole–Cole model [41]. Equation (5) [40] illustrates how a first-order model was used for fitting to simplify the model parameters. The electrical properties corresponding to variations in glucose concentration are shown in Figure 6 at different frequencies.

Figure 6 shows that both the real permittivity and conductivity decrease with increasing glucose concentration at 32 GHz and 42 GHz due to reduced water polarization and ionic mobility. In contrast, as frequency increases from 60 to 100 GHz, the relative permittivity decreases while conductivity increases because of dielectric relaxation and higher electromagnetic absorption. These frequency-dependent dielectric variations confirm the suitability of the Cole–Cole model for analyzing blood tissue behavior and support the development of microwave and millimeter-wave sensors for non-invasive glucose monitoring.

### 3.2. Simulated Glucose Sensing Results

To assess the proposed sensor across natural human ear anatomy, the ear thickness was set to 4 mm in a parametric simulation analysis. The range of adult ear thicknesses that have been anatomically documented is covered in [42]. A detailed multilayer ear phantom model was developed to emulate the anatomical structure of the human ear. The model consists of skin, cartilage, and blood vessel layers with geometrical dimensions selected based on anatomical references. The dielectric properties of each layer at the operating frequency were incorporated into the electromagnetic simulations, and are summarized in Table 2.

The equation shown is a thickness-weighted average permittivity model. The effective dielectric constant is calculated as

(6) $$\varepsilon_{r}^{eff}=\frac{\varepsilon_{r}^{sub}d_{sub}+\varepsilon_{r}^{blood}d_{blood}+\varepsilon_{r}^{skin}d_{skin}+\varepsilon_{r}^{fat}d_{fat}}{d_{sub}+d_{blood}+d_{skin}+d_{fat}}.$$

Thus, the effective dielectric constant would be ≈23.

**Figure 7:**
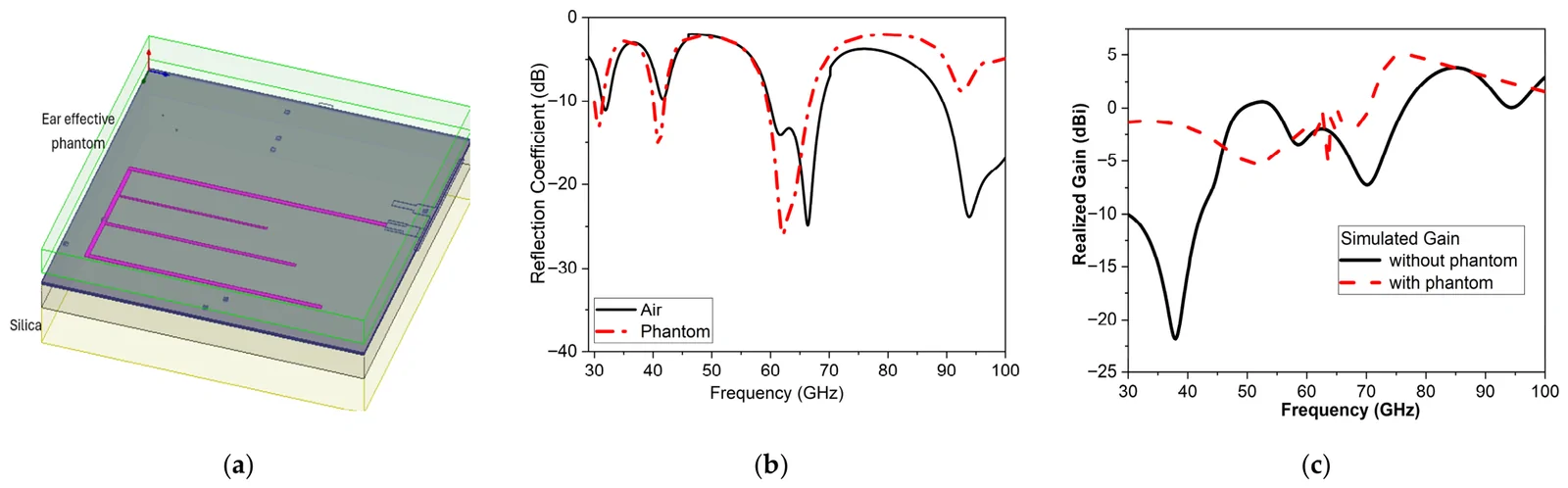
(**a**) Sensor configuration of the ear phantom. (**b**) |S11| magnitude with and without phantom. (**c**) The antenna gain in the presence of the ear phantom.

The simulated performance of the proposed mm-wave sensor in the presence of an ear phantom is shown in Figure 7. The practical near-body operating environment of the suggested system is illustrated by Figure 7a, which shows the geometrical configuration of the sensor integrated with the effective ear phantom model. The reflection coefficient |S_11_| responses with and without the phantom are contrasted in Figure 7b. Due to dielectric loading and electromagnetic interaction between the biological tissue and the antenna structure, the results demonstrate resonant frequency changes. The sensor retains good impedance matching over the specified working bands despite a minor resonance displacement, indicating reliable functioning under genuine biological settings. Figure 7c illustrates the simulated gain of the proposed antenna sensor, which is evaluated both without and with a phantom (tissue-mimicking material) over the frequency range of 30–100 GHz. In the air, without the phantom, the antenna exhibits multiple resonant behaviors with noticeable gain fluctuations across the spectrum. The realized gain reaches approximately 5 dBi near 100 GHz with an average gain of 2.5 dBi, while significant gain reductions are observed around 34 and 64 GHz due to impedance mismatch and resonant losses. After introducing the phantom, the gain response becomes smoother and more stable over a wide frequency range, although the maximum gain is slightly reduced. This reduction is mainly attributed to the dielectric loading and electromagnetic absorption introduced by the phantom material, which increases propagation losses and modifies the antenna radiation characteristics. Furthermore, the phantom shifts several resonant responses and reduces the sharp gain observed in free-space conditions. Despite the additional losses caused by the biological phantom, the sensor maintains acceptable realized gain performance across the mm-wave band, confirming its suitability for near-body and biomedical sensing applications such as non-invasive glucose monitoring.

For wearable sensing devices, evaluating human exposure to electromagnetic radiation is essential because a portion of the transmitted energy may be absorbed by surrounding biological tissues. The extent of this absorption depends on the dielectric properties of the tissues, the operating frequency, and the input power level. Therefore, the specific absorption rate (SAR) is commonly used to assess the safety of wearable sensors. According to IEEE C95.1-1999 and IEEE C95.1-2005 guidelines, the maximum permissible SAR limits are 1.6 W/kg averaged over 1 g of tissue and 2 W/kg averaged over 10 g of tissue [43].

In this study, the SAR was evaluated using the multilayer ear phantom model consisting of skin, fat, cartilage, and blood layers. The calculations were performed in HFSS using the 1 g averaged SAR method at the sensor resonant frequencies of 32, 42, 64, and 94 GHz for input power levels ranging from 0 to 15 dBm. The upper power level of 15 dBm was selected to represent a conservative worst-case operating condition and to provide an additional safety margin for practical wearable applications.

The results presented in Table 3 indicate that the SAR increases with increasing input power due to greater electromagnetic energy absorption within the biological tissues. The maximum SAR value obtained was 1.43 W/kg at 32 GHz and 15 dBm, which remains below the IEEE safety limit of 1.6 W/kg averaged over 1 g of tissue. Furthermore, all calculated SAR values satisfy both the previously cited IEEE C95.1-1999/2005 requirements and the limits specified in current international exposure guidelines. These findings confirm that the proposed sensor can operate safely within the investigated frequency range and is suitable for wearable, non-invasive biomedical applications, including continuous glucose monitoring.

**Figure 8:**
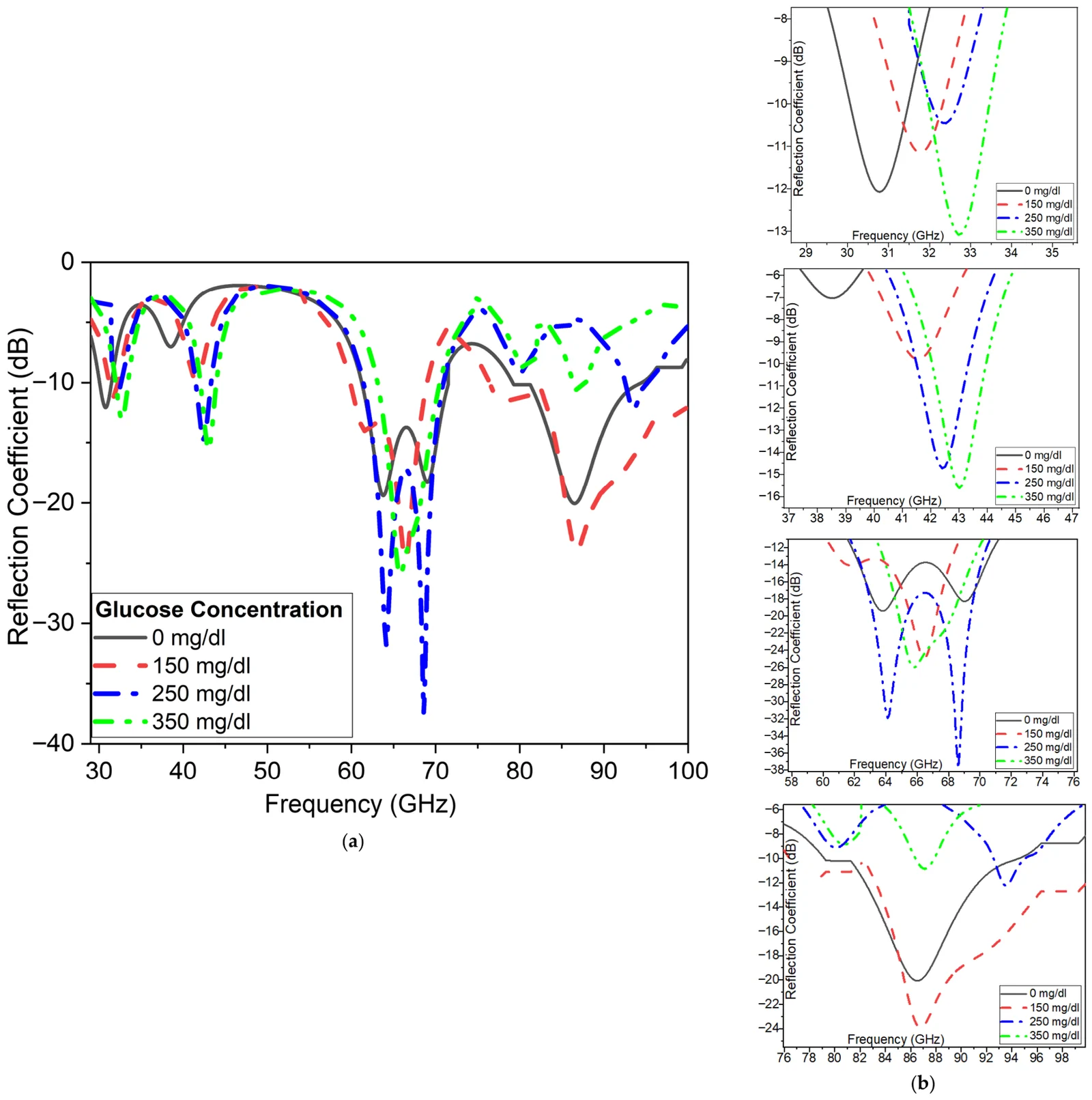
Simulation of different glucose concentrations: (**a**) reflection coefficient and (**b**) magnified zoom at selected frequencies.

Multiple resonance notches are observed across the 30–100 GHz range, as shown in Figure 8. As the glucose concentration increases, each resonance notch shifts toward lower frequencies, and the depth of the notch |S_11_| typically increases. Figure 8 illustrates the simulated |S_11_| of the proposed sensor for glucose concentrations of 0, 150, 250, and 350 mg/dL, representing the full-range response. Figure 8a shows multiple distinct resonance dips, indicating that the sensor operates effectively across a wide frequency band.

The zoomed views in Figure 8b highlight four specific resonance regions centered around ~37–47 GHz, ~58–76 GHz, ~76–98 GHz, and a higher-frequency feature. As the glucose concentration increases, each resonance exhibits a clear frequency shift, demonstrating the sensor’s sensitivity to changes in the dielectric properties of the glucose solution.

Table 3 quantifies the frequency shifts (Δf) at the four resonances for three glucose levels. The shifts are non-uniform across resonances: for example, at 350 mg/dL, the second resonance shows a Δf of 4500 MHz, while the fourth resonance reaches 6000 MHz, indicating that higher-order resonances may offer greater sensitivity. The average ΔS_11_ values (e.g., 1.5/8/12/10 dB at 250 mg/dL) confirm that the reflection coefficient amplitude also changes consistently with glucose concentration, further validating the sensor’s response.

Table 4 provides quantitative sensitivity metrics. It lists the absolute frequency shifts Δ*f* at four distinct resonances for glucose concentrations of 100, 250, and 350 mg/dL (likely relative to a reference, e.g., 0 mg/dL). The second resonance shows an Δ*f* of 4 GHz at 100 mg/dL, 5 GHz at 250 mg/dL, and 7 GHz at 350 mg/dL, providing a progressive increase that indicates near-linear sensitivity. The fourth resonance exhibits the most dramatic change, from Δf = 0.5 GHz at 100 mg/dL to 8 GHz at 350 mg/dL, corresponding to a sensitivity of approximately 0.03 GHz per mg/dL over that range.

Furthermore, the average change in |S_11_| magnitude rises from 1 dB at 100 mg/dL to 10 dB at 350 mg/dL, showing that the sensor not only shifts in frequency but also significantly alters return loss with increasing glucose. These quantitative values confirm that the sensor is highly sensitive to glucose variations across a clinically relevant range, with both frequency and amplitude-based sensing mechanisms available for reliable detection.

### 3.3. Measurement of the Proposed Sensor

**Figure 9:**
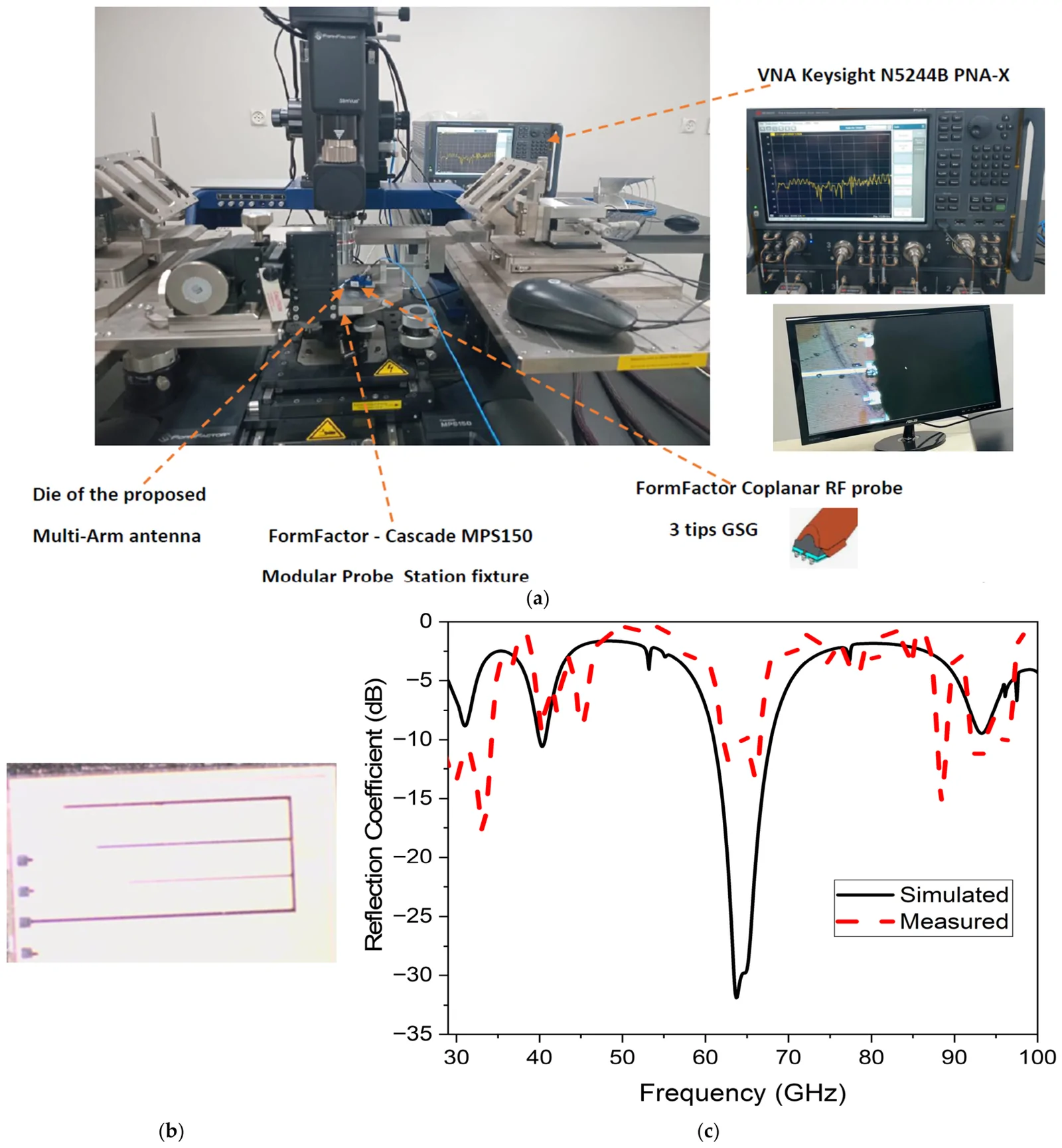
(**a**) Photo of the measurement’s setup. (**b**) Photo of fabricated sensor UMC mini@sic die. (**c**) Comparison of simulated and measured |S11| values.

The proposed multi-arm sensor reflection coefficient |S_11_| was measured at the Millimeter Waves Lab at the Electronics Research Institute (ERI). The measurement’s setup is shown in Figure 9, with the fabricated die of the proposed sensor fixed on the fixture base of the Cascade MPS150 Modular Probe Station. A FormFactor Coplanar RF probe is used for direct contact with the fabricated die pads, with a three-tipped GSG. The measurements were performed using a Keysight N5244B PNA-X vector network analyzer and a Keysight N5293AX01 broadband frequency extender (Keysight Technologies, Inc., Santa Rosa, CA, USA), which extends the measurement frequency range up to 110 GHz.

## 4. Discussion

The use of wearable sensors with multiple arms in in vitro tests to differentiate between various glucose concentrations in diabetic patients with hypoglycemia, normoglycemia, and hyperglycemia is demonstrated in this paper. To find out how changes in glucose concentration impact the reflection coefficient—which was used to calculate the sensor’s sensitivity—the frequency band is adjusted and tested over a range. The resonant frequencies for the relative permittivity ε_r_ and the sensor’s sensitivity are altered when blood is introduced due to the interaction between the blood glucose samples and the fields. The sensitivity is analyzed using Equation (7), which demonstrates that a change in the glucose concentration (ΔC) determines any change in the sensor’s resonant frequency (Δf) or magnitude |ΔS_11_|. Equations (7) and (8) can be extracted, as in [20].

(7) $$S_{fr}=\frac{∆F}{∆C}MHz/mg/dL.$$

(8) $$S_{dB}=\frac{∆S}{∆C}dB/mg/dL.$$

In terms of sensitivity and size, sensitivity is concentration-dependent and varies significantly among resonances, indicating that optimal operating points can be selected based on the target glucose range. Two sensitivity metrics are reported in Table 5: the first set of values, 9, 6.4, and 7.4 MHz/mg/dL for the 1st resonance, and the second set consisting of 0.01, 0.006, and 0.003 dB/mg/dL. The second and fourth resonances exhibit the highest sensitivities, with the second resonance reaching 30 at 100 mg/dL and 14–13 at higher concentrations, while the fourth resonance shows 26 at 250 mg/dL. The third resonance displays the lowest sensitivity at 250 mg/dL (value of 2). This confirms that the second and fourth resonances are more responsive to glucose changes.

The resonant response does not exhibit a strictly monotonic dependence on glucose concentration at all operating frequencies. This behavior is attributed to the frequency-dependent dielectric dispersion of biological tissues, particularly blood, whose effective permittivity and conductivity vary differently across the investigated frequency spectrum. Consequently, individual resonances may exhibit varying trends in frequency shift and reflection coefficient response. Therefore, the sensing mechanism should be interpreted as a multifrequency dielectric signature rather than a single monotonic resonance shift.

Table 6 compares the proposed sensor’s performance with that presented in the literature. Recent advances in microwave and millimeter-wave sensing technologies have demonstrated significant potential for biomedical diagnostics and non-invasive glucose monitoring. These sensing systems mainly operate using either reflection-mode or transmission-mode techniques, each offering distinct advantages in sensitivity, compactness, and implementation complexity.

An on-chip antenna in [38] was implemented as a dual-meander line using two metal layers backed by a ground sheet, targeting a 0.18 μm CMOS technology, for biomedical applications. The designed antenna offered multi-band operation at four frequency bands (22/34/44/58 GHz), consuming a die area of 250 μm ×1525 μm. The multifrequency band operation offered the possibility of reserving the lowest band for power transfer, the 44 GHz band for cancer detection, while the 34/58 GHz bands were allocated for data transfer.

A wideband on-chip dipole antenna was demonstrated in [44]. The antenna was implemented in a 0.18 μm CMOS technology, comprising a dipole radiator on the upper metal layer, a patch added in an intermediate layer, and a ground sheet in the bottom metal layer. The proposed antenna consumed a die area of 130 × 250 μm^2^, offering wide band operation from 21 to 40 GHz. For glucose detection using mm waves, several off-chip, rather than on-chip, solutions were proposed. In [45], a ready module for 60 GHz radar was used. Most existing designs operate at single frequency bands (e.g., 22–58 GHz in [38], 58–63.5 GHz in [45]) and utilize reflection- or transmission-based techniques, with gains ranging from −20 dBi to 7.77 dBi. Notably, only a few previous works [38,44] are implemented on-chip, whereas the proposed sensor is fully on-chip with a compact area of 0.9 mm^2^. In terms of sensitivity, the proposed sensor achieves 12.4 MHz/(mg/dL) and 6 dB/(mg/dL), outperforming or competing with reported values such as 10 mg/dL in [45] or 0.027 mg/dL in [46].

Furthermore, the proposed multi-arm antenna design operates at four distinct resonances (32, 42, 64, and 94 GHz) with a gain of 2.5 dBi, offering multifrequency glucose detection. Crucially, specific absorption rate (SAR) simulations confirm that the proposed design complies with international safety standards, yielding a maximum SAR value well below the regulatory limit of 2 W/kg (averaged over 10 g of tissue), making it suitable for continuous or repeated biomedical use.

Although the proposed multifrequency millimeter-wave CMOS sensor demonstrates promising sensitivity to glucose-induced dielectric variations, the current study is limited to simulation-based glucose monitoring. Future work will focus on experimental validation using glucose-controlled tissue phantoms, liquid glucose solutions, and, ultimately, human-subject studies. In addition, further work will address packaging, calibration, wearable integration, and long-term stability to realize a practical, non-invasive glucose monitoring system. Approval from the Ethical Perspective Committee (NILES-EC-CU 24/1/2) was obtained in August 2024. Upon completion of the system integration phase, subsequent studies will be conducted involving both healthy and diabetic participants to assess the system’s performance and applicability.

## 5. Conclusions

In this paper, a small, multifrequency mm-wave sensor for non-invasive continuous glucose monitoring was developed using UMC 0.18 μm CMOS technology. By operating at four different frequencies—32, 42, 64, and 94 GHz—the proposed multi-arm antenna allows for multi-band sensing that takes advantage of the frequency-dependent dielectric characteristics of human tissues. Both the |S_11_|and realized gain were assessed both with and without tissue loading by integrating the sensor with an anatomically accurate ear phantom. According to simulation results, the ear phantom smooths gain response by an average of 2.5 dBi while maintaining acceptable radiation performance, and the sensor maintains, at the same time, stable impedance matching |S_11_| ≤ −6 dB across the operating bands. The proposed sensor was used to design, construct, and assess a non-invasive glucose monitoring method that precisely simulates the location of blood vessels in the ear phantom. Even at the maximum input power of 15 dBm, SAR analysis verifies that all simulated values are within IEEE safety guidelines, guaranteeing safe operation for wearable applications. The fourth resonance shows a frequency shift of up to 8 GHz and an average change of 10 dB at 350 mg/dL. Systematic modulation of glucose concentration (0–350 mg/dL) results in distinct, monotonic shifts in resonant frequencies and increases in |S_11_| magnitude. High sensitivity and superior discrimination ability across clinically relevant glucose levels are demonstrated by these quantitative measurements. The proposed design is a promising solution for future wearable, real-time, and non-invasive glucose monitoring systems because it has the advantages of full on-chip integration, a compact area of 0.9 mm^2^, multi-band operation, and competitive sensitivity when compared to earlier off-chip glucose sensors.

## Figures and Tables

**Table 1:** The dimensions of the proposed sensor; all dimensions in micrometers (μm).

***W_Sub_***	***L_Sub_***	***W_f_*** **_1_**	***L*** **_1_**	***L_f_*** **_1_**	***W_f_*** **_2_**
1300	700	20	620	175	15
***W*** **_1_**	***W*** **_2_**	***W*** **_3_**	***W*** **_4_**	***L_f_*** **_2_**	***W_f_*** **_3_**
1220	700	900	1100	195	10

**Table 2:** Electrical properties of ear phantom layers at frequency 20 GHz.

**Parameters**	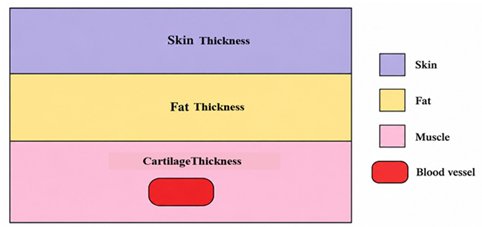	**Thickness**	$\varepsilon_{r}$	σ	**Tan δ**
**Skin**		1 mm	38.0	1.46	0.28
**Fat**		0.5 mm	5.28	0.10	0.13
**Cartilage**		2 mm	42.8	1.69	0.28
**Blood**		0.5 mm	58.0	2.54	0.32

**Table 3:** The simulated SAR levels for the proposed sensor.

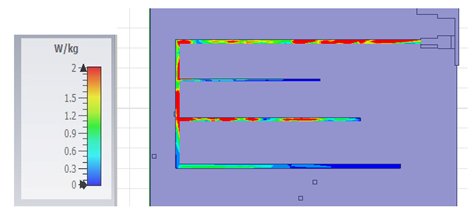									
	Fre. (GHz)	32		42		64		94	
P (dBm)		1 g (W/Kg)	10 g (W/Kg)	1 g(W/Kg)	10 g (W/Kg)	1 g (W/Kg)	10 g (W/Kg)	1 g (W/Kg)	10 g (W/Kg)
**0**		0.19	0.1 W/Kg	0.1	0.08	0.01	0.15	0.131	0.032
**5**		0.84	0.27 W/Kg	0.54	0.2	0.087	0.42	0.181	0.045
**10**		1.13	0.55	0.831	0.35	0.273	0.131	0.231	0.068
**15**		1.43	0.879	1.13	0.879	0.851	0.410	0.738	0.217

**Table 4:** The proposed sensor effect with variations in glucose concentration.

Glucose Concentration	100 mg/dL	250 mg/dL	350 mg/dL
$∆f\left(MHz\right)$ in 1st resonance	900	1600	2600
$∆f\left(MHz\right)$ in 2nd resonance	3000	3500	4500
$∆f\left(MHz\right)$ in 3rd resonance	1500	500	1500
$∆f\left(MHz\right)$ in 4th resonance	500	6500	6000
**Average** $∆S11\left(dB\right)$	1/3/6/4	1.5/8/12/10	1.5/9/6/11

**Table 5:** Calculation of sensitivity at different frequencies.

Concentration mg/dL	Equation (7)			Equation (7)		
	100	250	350	100	250	350
**1st Resonance**	9	6.4	7.4	0.01	0.006	0.003
**2nd Resonance**	30	14	13	0.03	0.032	0.018
**3rd Resonance**	15	2	4.2	0.06	0.048	0.012
**4th Resonance**	5	26	17	0.04	0.04	0.022

**Table 6:** Comparison between proposed sensors and other published sensors in the literature.

Ref.	Sensing Technique	Application	Antenna	On-Chip/ML	Gain (dB)	Frequency Band(s) (GHz)	Sensitivity	Range mg/dL	Area mm^2^
**[38]**	Reflection	Biomedical	Dual-meander line	Yes/No	−1/−10/−15/−20	22/34/44/58	-	-	0.23
**[44]**	Reflection	Soil moisture	Dipole + patch	Yes/No	−2	26–37	-	-	0.325
**[45]**	Reflection	Glucose	U-slotted patch antenna	No/Yes	3	58–63.5	10 mg/dL	50–200	-
**[46]**	Reflection	Glucose	Five split-rings	No/No	-	24.9	0.027 dB/mg/dL (sim.)	70–1000	600
**[47]**	Transmission	Glucose	Two facing microstrip patches	No/	7.77 dBi	60	0.00025 dB/mg/dL	25–4000	2.25
**[48]**	Transmission	Glucose	Directional coupler	No/Yes	-	75–111	100% correct (Healthy/Diab.)	100–300	-
**[14]**	Transmission	Glucose	Two opposingly facing patch antennas	No/Yes	-	37–39	0.00026 dB/(mg/dL)	80–5000	-
**Proposed**	Reflection	Glucose	Multi-arm	Yes/No	2.5	32/42/64/94	12.4 MHz/mg/dL6 dB/mg/dL(sim.)	0–350	0.9

ML: Post-processing by machine learning. sim.: Denotes simulated result.

## Data Availability

The data that may be required to reproduce the findings of this study are available upon reasonable request from the corresponding author.

## References

[1] Nakrani M.N., Wineland R.H., Anjum F. (2022). Physiology, Glucose Metabolism.

[2] Farmaki P., Damaskos C., Garmpis N., Garmpi A., Savvanis S., Diamantis E. (2020). Complications of the Type 2 Diabetes Mellitus. Curr. Cardiol. Rev..

[3] Shah N.S., Wang M.C., Freaney P.M., Perak A.M., Carnethon M.R., Kandula N.R., Gunderson E.P., Bullard K.M., Grobman W.A., O’Brien M.J. (2021). Trends in Gestational Diabetes at First Live Birth by Race and Ethnicity in the US, 2011–2019. JAMA.

[4] McIntyre H.D., Catalano P., Zhang C., Desoye G., Mathiesen E.R., Damm P. (2019). Gestational Diabetes Mellitus. Nat. Rev. Dis. Primers.

[5] International Diabetes Federation (2021). IDF Diabetes Atlas.

[6] Sami W., Ansari T., Butt N.S., Ab Hamid M.R. (2017). Effect of Diet on Type 2 Diabetes Mellitus: A Review. Int. J. Health Sci..

[7] Tang L., Chang S.J., Chen C.-J., Liu J.-T. (2020). Non-Invasive Blood Glucose Monitoring Technology: A Review. Sensors.

[8] Delbeck S., Heise H.M. (2021). Evaluation of Opportunities and Limitations of Mid-Infrared Skin Spectroscopy for Noninvasive Blood Glucose Monitoring. J. Diabetes Sci. Technol..

[9] Bader H.D., Jarjees M.S., Ahmed B.T. (2023). Invasive and non-invasive glucose monitoring systems: A review and comparative study. Prz. Elektrotech..

[10] Althobaiti M., Al-Naib I. (2021). Optimization of dual-channel near-infrared non-invasive glucose level measurement sensors based on Monte-Carlo simulations. IEEE Photonics J..

[11] Joshi A.M., Jain P., Mohanty S.P., Agrawal N. (2020). iGLU 2.0: A new wearable for accurate non-invasive continuous serum glucose measurement in IoMT framework. IEEE Trans. Consum. Electron..

[12] Kang J.W., Park Y.S., Chang H., Lee W., Singh S.P., Choi W., Galindo L.H., Dasari R.R., Nam S.H., Park J. (2020). Direct observation of glucose fingerprint using in vivo Raman spectroscopy. Sci. Adv..

[13] Abdel-Haleem M.R., Afifi A.I., Abd El-Hameed A.S. (2026). Non-invasive blood glucose sensing with a wearable microwave antenna earring. Sens. Actuators A Phys..

[14] Cano-Garcia H., Kshirsagar R., Pricci R., Teyeb A., O’Brien F., Saha S., Kosmas P., Kallos E. (2021). Enhancing the Accuracy of Non-Invasive Glucose Sensing in Aqueous Solutions Using Combined Millimeter Wave and Near Infrared Transmission. Sensors.

[15] Kasahara R., Kino S., Soyama S., Matsuura Y. (2018). Noninvasive Glucose Monitoring Using Mid-Infrared Absorption Spectroscopy Based on a Few Wavenumbers. Biomed. Opt. Express.

[16] Han T., Liu X., Liu J., Xu K. (2019). An Optimized Non-Invasive Glucose Sensing Based on Scattering and Absorption Separating Using Near-Infrared Spectroscopy. Proc. SPIE.

[17] Yu Z.F., Pirnstill C.W., Coté G.L. (2016). Dual-Modulation, Dual-Wavelength, Optical Polarimetry System for Glucose Monitoring. J. Biomed. Opt..

[18] Elsibaie S.S., Eldamak A.R., Elsheakh D.N. (2026). Comparative study of Complementary Split Ring Resonator (CSRR) designs for non-invasive blood glucose monitoring using microwave sensors. J. Electromagn. Waves Appl..

[19] Guo W.T., Zahran A., El-Shal I.H., Guo Y., Eldamak A.R., Fahmy O.M., Yuan Z., Tai H., Elsheakh D.N., Lin Y. (2026). Multimodal biosensing platforms for sensor-computing integration towards intelligent healthcare. Soft Sci..

[20] Farouk M., El-Hameed A.S.A., Eldamak A.R., Elsheakh D.N. (2025). Noninvasive blood glucose monitoring using a dual band microwave sensor with machine learning. Sci. Rep..

[21] Elsheakh D.N., Mohamed E.-H., Eldamak A.R. (2025). Blood Glucose Monitoring Biosensor Based on Multiband Split-Ring Resonator Monopole Antenna. Biosensors.

[22] Lee S., Kim K., Yang Y., Seong J., Jung C., Lee H.J., Rho J. (2024). Deep learning-driven robust glucose sensing and fruit brix estimation using a single microwave split ring resonator. Laser Photonics Rev..

[23] Bakogianni S., Tsolis A., Angelaki C., Alexandridis A.A. (2024). On the Development of Embroidered Reconfigurable Dipole Antennas: A Textile Approach to Mechanical Reconfiguration. Electronics.

[24] Liu F.-X., Meng F.-Y., Chen Y.-J., Gao Z.-H., Cui J., Zhang L. (2024). Textile Bandwidth-Enhanced Half-Mode Substrate-Integrated Cavity Antenna Based on Embroidered Shorting Vias. Micromachines.

[25] Du C.-Z., Yang F.-H., Zhong S.-S. Dual-Polarized Textile Antenna Integrated in the Three-Dimensional Orthogonal Woven Fabrics. Proceedings of the 2015 IEEE MTT-S International Microwave Workshop Series on Advanced Materials and Processes for RF and THz Applications (IMWS-AMP).

[26] Nguyen T.M., Chung J.-Y., Lee B. (2015). Radiation Characteristics of Woven Patch Antennas Composed of Conductive Threads. IEEE Trans. Antennas Propag..

[27] Boulerbah R., Chaabane A., Attia H., Aissaoui D. Breast Cancer Detection Using Circularly Polarized Printed UWB Antenna. Proceedings of the 2nd International Conference on Electronics, Energy and Measurement (IC2EM).

[28] Raj S., Tripathi S., Upadhyay G., Tripathi S.S., Tripathi V.S. (2021). An Electromagnetic Band Gap-Based Complementary Split Ring Resonator Loaded Patch Antenna for Glucose Level Measurement. IEEE Sens. J..

[29] Yilmaz T., Foster R., Hao Y. (2014). Broadband Tissue Mimicking Phantoms and a Patch Resonator for Evaluating Noninvasive Monitoring of Blood Glucose Levels. IEEE Trans. Antennas Propag..

[30] Garcia-Pardo C., Antonino-Daviu E., Castelló-Palacios S., Vila-Jimenez A., Vallés-Lluch A., Cardona N. 60 GHz Wearable Flexible Antenna in a Customized Multilayer Body Phantom. Proceedings of the 33rd Annual International Symposium on Personal, Indoor and Mobile Radio Communications (PIMRC).

[31] Castelló-Palacios S., Garcia-Pardo C., Alloza-Pascual M., Fornes-Leal A., Cardona N., Vallés-Lluch A. (2019). Gel Phantoms for Body Microwave Propagation in the (2 to 26.5) GHz Frequency Band. IEEE Trans. Antennas Propag..

[32] ICNIRP (2020). Guidelines for Limiting Exposure to Electromagnetic Fields (100 kHz to 300 GHz).

[33] (2015). Vitro Diagnostic Test Systems—Requirements for Blood-Glucose Monitoring Systems for Self-Testing in Managing Diabetes Mellitus.

[34] Gharbi M.E., Fernández-García R., Gil I. (2021). Textile Antenna-Sensor for In Vitro Diagnostics of Diabetes. Electronics.

[35] Hanna J., Costantine J., Kanj R., Tawk Y., Ramadan A.H., Eid A.A. A Vasculature Anatomy Inspired Flexible Slot Antenna for Continuous Non-invasive Glucose Monitoring. Proceedings of the IEEE International Symposium on Antennas and Propagation and USNC-URSI Radio Science Meeting (APS/URSI).

[36] Di Filippo D., Sunstrum F.N., Khan J.U., Welsh A.W. (2023). Non-Invasive Glucose Sensing Technologies and Products: A Comprehensive Review for Researchers and Clinicians. Sensors.

[37] Yunos M.F.A.M., Manczak R., Guines C., Mansor A.F.M., Mak W.C., Khan S., Ramli N.A., Pothier A., Nordin A.N. (2021). RF Remote Blood Glucose Sensor and a Microfluidic Vascular Phantom for Sensor Validation. Biosensors.

[38] Shawkey H., Elsheakh D. (2020). Multiband Dual-Meander Line Antenna for Body-Centric Networks’ Biomedical Applications by Using UMC 180 nm. Electronics.

[39] Qin Y., Zhang L., Han T., Liu Y., Liu X., Fu F., Wang H., Qu S., Zhao Z., Yang L. (2025). An Improved Cole–Cole Model for Characterizing In Vivo Dielectric Properties of Lung Tissue at Different Tide Volumes: An Animal Study. Bioengineering.

[40] Etoz S., Brace C.L. (2019). Development of Water Content Dependent Tissue Dielectric Property Models. IEEE J. Electromagn. RF Microw. Med. Biol..

[41] Mattana E., Lodi M.B., Simone M., Mazzarella G., Fanti A. (2024). Cole–Cole Model for the Dielectric Characterization of Healthy Skin and Basal Cell Carcinoma at THz Frequencies. IEEE Open J. Eng. Med. Biol..

[42] Kim J., Kim B.K., Park M.-R., Cho H., Huh C. (2025). Noninvasive continuous glucose monitoring using multimodal near-infrared, temperature, and pressure signals on the earlobe. Biosensors.

[43] Sharaf F., Elsheakh D.N., Eldamak A.R. (2025). A Noninvasive Method of Monitoring Blood Glucose Levels by Using Triple-Band Monopole Antenna. Microw. Opt. Technol. Lett..

[44] Elsheakh D., Shawkey H. (2021). 5G wideband on-chip dipole antenna for WSN soil moisture monitoring. Int. J. RF Microw. Comput.-Aided Eng..

[45] Nikandish R., Sheedy C., He J., Crowe R., Rao D. (2025). Contactless Glucose Sensing Using Miniature mm-Wave Radar and Tiny Machine Learning. IEEE J. Microw..

[46] Qureshi S.A., Zainal Abidin Z., Elamin N.I.M., Majid H.A., Ashyap A.Y.I., Nebhen J., Kamarudin M.R., See C.H., Abd-Alhameed R.A. (2022). Glucose level detection using millimetre-wave metamaterial-inspired resonator. PLoS ONE.

[47] Saha S., Cano-Garcia H., Sotiriou I., Lipscombe O., Gouzouasis I., Koutsoupidou M., Palikaras G., Mackenzie R., Reeve T., Kosmas P. (2017). A Glucose Sensing System Based on Transmission Measurements at Millimetre Waves using Microstrip Patch Antennas. Sci. Rep..

[48] Moreno-Oyervides A., Martín-Mateos P., Aguilera-Morillo M.C., Ulisse G., Arriba M.C., Durban M., Del Rio M., Larcher F., Krozer V., Acedo P. (2019). Early, Non-Invasive Sensing of Sustained Hyperglycemia in Mice Using Millimeter-Wave Spectroscopy. Sensors.

